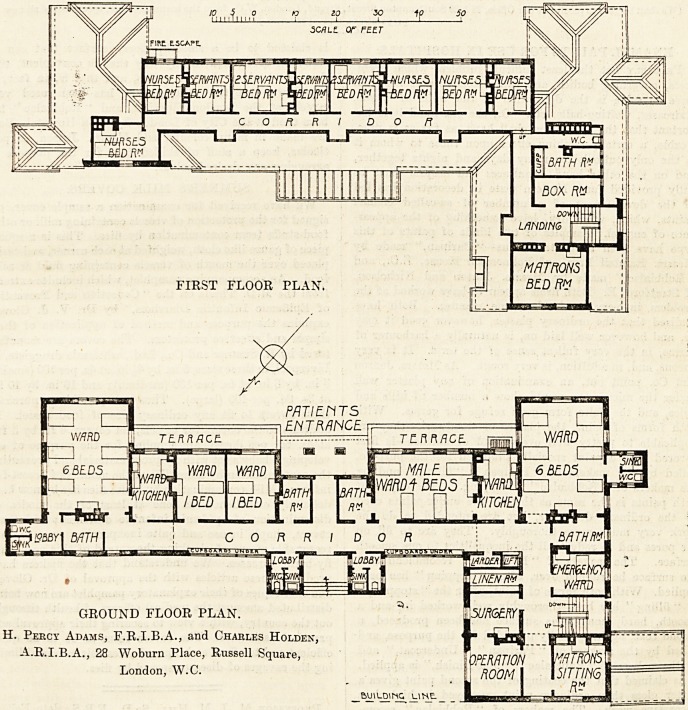# Southport Homœopathic Hospital

**Published:** 1909-06-26

**Authors:** 


### HOSPITAL ADMINISTRATION.

### CONSTRUCTION AND ECONOMICS.

### SOUTHPORT HOMŒOPATHIC HOSPITAL.

THIS small hospital has been built on the site of an old battery on the sand hills, and the irregular form of the ground has considerably influenced the plan. The building consists of three floors, namely, a basement under the whole of the west wing, and part of the east wing, with a subway for pipes connecting the two, ground floor, and upper floor over part of the ground floor.

FIRST FLOOR PLAN.

GROUND FLOOR PLAN.

H. PERCY ADAMS, F.R.I.B.A., and CHARLES HOLDEN, A.R.I.B.A., 28 Woburn Place, Russell Square, London, W.C.

The west wing of the basement which is at the ground level on the south-west side contains the kitchen offices, entrance for staff, staff dining-room and heating apparatus. The basement under the east wing contains quarters for a married couple, and an ambulance house.

The ground floor, the main front of which faces south-east, contains two wards for six beds, one for four; an emergency ward for one bed and two single wards presumably for paying patients. As the four-bed ward alone is definitely labelled "Male," we presume the other two large wards are to be used for male or female patients as occasion may arise.

There are no less than four bath-rooms on this floor, three of which open direct into the wards—an excellent arrangement, and much better than the plan of putting bath-rooms into projecting towers with intervening lobbies which are not required; but the provision of so many bath-rooms seems somewhat liberal, if not excessive.

There are two ward kitchens, and four sets of sanitary offices. The sink-rooms are much too small for the work that has to be done in them, and the sanitary spur to the south-west large ward is a serious obstacle to the ventilation of the ward. In fact, the planning of the wards cannot be regarded as satisfactory, seeing that in each case one side wall of the ward along which three beds are placed has only one window. This is not the sort of planning which we expect from an architect of Mr. Percy Adams' experience.

The operation-room, with surgery adjoining, is placed in the north-west wing and faces north-east, and immediately facing it is the matron's sitting-room, not a particularly happy arrangement for the matron. The upper floor contains the matron's bedroom, and rooms for four nurses and six servants, with a box-room and one bath-room to be used in common, we presume, by the matron and servants. It would surely have been better to have provided one bath-room for the matron and nurses, and one for the servants, and to have omitted one of the four bath-rooms for patients.

The whole cost of the building, including road making, laying-out grounds, and boundary fences, etc., amounted to the sum of £4,490—or a little over £236 per bed. A remarkably small sum, which seems to show that building must be very cheap in Southport. The architects were Messrs. Percy Adams and Holden.

## Figures and Tables

**Figure f1:**